# Breeding with resistant rams leads to rapid control of classical scrapie in affected sheep flocks

**DOI:** 10.1186/1297-9716-42-5

**Published:** 2011-01-11

**Authors:** Gonnie Nodelijk, Herman JW van Roermund, Lucien JM van Keulen, Bas Engel, Piet Vellema, Thomas J Hagenaars

**Affiliations:** 1 Department of Epidemiology, Crisis organisation and Diagnostics, Central Veterinary Institute of Wageningen UR, P.O. Box 65, 8200 AB Lelystad, the Netherlands; 2 Department of Bacteriology and TSEs, Central Veterinary Institute of Wageningen UR, P.O. Box 65, 8200 AB Lelystad, the Netherlands; 3 Animal Health Service, P.O. Box 9, 7400 AA Deventer, the Netherlands

## Abstract

Susceptibility to scrapie, a transmissible spongiform encephalopathy in sheep, is modulated by the genetic make-up of the sheep. Scrapie control policies, based on selecting animals of resistant genotype for breeding, have recently been adopted by the Netherlands and other European countries. Here we assess the effectiveness of a breeding programme based on selecting rams of resistant genotype to obtain outbreak control in classical scrapie-affected sheep flocks under field conditions. In six commercially-run flocks following this breeding strategy, we used genotyping to monitor the genotype distribution, and tonsil biopsies and post-mortem analyses to monitor the occurrence of scrapie infection. The farmers were not informed about the monitoring results until the end of the study period of six years. We used a mathematical model of scrapie transmission to analyze the monitoring data and found that where the breeding scheme was consistently applied, outbreak control was obtained after at most four years. Our results also show that classical scrapie control can be obtained before the frequency of non-resistant animals is reduced to zero in the flock. This suggests that control at the national scale can be obtained without a loss of genetic polymorphisms from any of the sheep breeds.

## Introduction

Classical scrapie in sheep is the eldest known transmissible spongiform encephalopathy (TSE) and is present in all sheep-producing countries except Australia and New Zealand [1,2]. Infection mostly occurs at very young age and clinical signs of this fatal disease are visible after a variable incubation period of one or more years dependent of genotype [3]. Frequently observed clinical signs are uncoordinated movement (ataxia), abnormal posture and severe scratching and rubbing. During the incubation period the prion protein PrP^Sc ^slowly accumulates in the animal and can be detected for most sheep genotypes in lymphoid organs such as tonsils before clinical signs become visible [4,5].

Scrapie control became a priority in many countries some ten years ago. This was motivated in part by the theoretical possibility that bovine spongiform encephalopathy (BSE) may in the past have been introduced into sheep through consumption of feed supplements, with potential consequences to public health [6,7]. This possibility became apparent after experimental infection of sheep with BSE showed that sheep can be infected via the oral route and that the resulting clinical symptoms are very similar to scrapie [8,9].

The susceptibility to scrapie is modulated by polymorphisms of the sheep prion protein (PrP) gene. For classical scrapie strains the most relevant polymorphisms occur at codons 136, 154 and 171 of the PrP gene [10-13], and for the recently discovered atypical scrapie an additional polymorphism has been identified at codon 141 [14-16]. For atypical strains the between-animal transmissibility in the field, if at all present, is likely to be very low [2].

In this study we focus on classical scrapie and thus on the three aforementioned codons. Four alleles (VRQ, ARQ, AHQ and ARR) were observed in this study, and each one of the corresponding 10 possible genotypes occurred in the study flocks. The VRQ allele confers high susceptibility to most strains of classical scrapie, the ARQ allele is associated with moderate susceptibility and the AHQ allele may be associated with increased resistance and longer incubation periods [17,18]. The allele ARR is known to confer resistance to all strains of classical scrapie, with the homozygote genotype ARR/ARR being extremely resistant, and the heterozygote ARQ/ARR and AHQ/ARR genotypes being only rarely affected by classical scrapie [19,20].

The current scrapie control regulation in the European Union consists of the following two minimum requirements: culling of all animals of susceptible genotype in infected flocks (if no genetic testing is done, all animals need to be culled), and the genetic testing of rams intended for breeding in scrapie-free flocks of "high genetic merit" followed by culling of the susceptible rams (EC Regulation No 999/2001 [21]). Several years before the EC scrapie control regulation came into force, some member states already had a national breeding programme that sheep breeders could join on a voluntary basis, notably the Netherlands (started in 1998), Great Britain (started in 2001) and France (started in 2001). In the Netherlands, the selection of resistant rams (ARR/ARR) for breeding was made compulsory for the sheep industry in November 2004.

The purpose of this study is to assess under field conditions how a breeding programme based on selecting rams of resistant genotype affects the transmission of classical scrapie. To this end we recruited six scrapie-affected commercially-run sheep flocks into our study that were willing to join the (initially voluntary) breeding programme. Both the genotype distribution and scrapie transmission in each of these flocks was monitored over a period of 4 to 6 years.

## Materials and methods

### Study population

Six commercially-run sheep farms (labelled from A to F) were selected in 2000 on the basis of the following criteria. We were interested in flocks with a recent history of (confirmed) scrapie cases, with individual identification and registration of the animals, with the perspective of continuation of the flock for at least five years, and with a limited sale of animals over six months of age. Furthermore, the farmer needed to be willing to take part in the research project for at least four years, and to use only ARR/ARR rams for breeding starting from, at the latest, the 2001 mating season until the end of the study period, and to purchase no animals except ARR/ARR rams during this period. The research project bought all live animals older than 12 months that were culled by the farmer. The farms kept breeding flocks with an average size of between 90 and 100 ewes. Flock A has a size of about 125 ewes (pure-bred Texel and Suffolks). In this flock scrapie had been confirmed in 1998, 1999 and 2000 in four sheep. Flock B and C both have a flock size of about 125 ewes (unregistered Texel). In flock B scrapie had been confirmed in 1997 and 1999 in four sheep, and flock C had about 10-15 unconfirmed scrapie-suspected sheep each year since 1992. Flock D has an average size of 50 ewes (cross-breeds), in which scrapie was confirmed in one sheep in 2000. Flock E has about 150 ewes (cross-breeds). Since 1997 there had been a few scrapie cases each year in this flock, with a peak of seven cases in the 1999-2000 winter, and scrapie was officially confirmed in 2000 in three sheep. Flock F has 35 Swifter ewes, and scrapie had been confirmed in 1998 and 2000 in four sheep. In total the study population consisted of about 550-600 sheep each year.

### Monitoring approach

Each flock in the study was monitored by carrying out the following three types of analysis:

1. Genotyping of all breeding animals older than 12 months at the start of the study and of all new breeding animals (own offspring and purchased rams) in subsequent years. PrP genotypes were determined (at least at codons 136, 154, and 171) by a routine TaqMan test [18] that is completely automated. A second test, based on sequencing, was used as a confirmatory test on random selected samples.

2. Yearly tonsil biopsy of all breeding sheep older than 12 months, and testing these biopsies for presence of PrP^Sc^, the pathogenic isoform of the prion protein [22,23]. The biopsy technique has been described in detail by Schreuder et al. [4].

3. Post-mortem testing for the presence of PrP^Sc ^in tonsils and brains of all animals older than 12 months that were culled or died of intercurrent disease on the farm. At necrosy, the brain stem was collected together with both palatine tonsils for histopathological examination [22,23].

The yearly sampling of blood and tonsils took place between May and September, after weaning of the lambs and before the mating season started. Of the genotyping and the test results, sheep owners only received the genotype of the rams, to be able to select resistant rams for breeding. No information about ewe genotypes or scrapie status of the animals was given during the entire project period.

Two of the six flocks (A, F) were monitored during 4 consecutive years and the remaining 4 flocks during 6 years. The monitoring length of six years was designed to be sufficient to monitor the vast majority of animals born before 2001 throughout their lives. For later cohorts the right-censoring of the data was expected to become increasingly important to take into consideration when interpreting these data.

### Assessment of scrapie cases

The presence of PrP^Sc ^was tested in the yearly tonsil biopsies and in the obex and tonsils collected at necropsy by histopathological examination [22,23]. Brain obex: a scrapie positive diagnosis was made when the typical pattern of PrP^Sc ^(intracellular and cell membrane staining of neurons and glial cells) was found in the obex. In those cases in which the dorsal motor nucleus of the Vagus (DMNV) was not available for examination (e.g. severe autolysis or inadequate sampling) no diagnosis was made and the sample was considered to be unsuitable. A negative diagnosis was made when the DMNV was available for examination but no PrP^Sc ^could be detected. Tonsil: a diagnosis of scrapie was made when the typical pattern of PrP^Sc ^(on the cell membrane of follicular dendritic cells and in follicular macrophages) was detected in the lymphoid follicles. When a tonsil biopsy contained <3 lymphoid follicles, it was considered unsuited for scrapie diagnosis. A negative diagnosis was made when the number of lymphoid follicles was ≥3 and no PrP^Sc ^could be detected.

Because testing for the presence of PrP^Sc ^is based on the monitoring procedure and not on the presence of clinical signs of scrapie, histopathological examination can also reveal subclinal scrapie. In this study a scrapie case is defined as a sheep with detected PrP^Sc ^in brain and/or tonsils independent of the presence of clinical signs.

All animals of the flock present at the yearly sampling were tested by tonsil biopsy (and occasionally by almost coincidental post-mortem examination). Scrapie *prevalence *was estimated for each year by the number of scrapie-positive tested animals divided by the number of tested animals. The prevalence can be underestimated as PrP^Sc ^will not be detected in tonsils for all susceptible genotypes (e.g. VRQ/ARR). If a sheep was culled within some weeks after the yearly sampling, the post-mortem result was used to compute the prevalence. The *incidence *rate was estimated by the number of new detected scrapie cases during a year following the last yearly sampling, divided by the number of animal-time units at risk. New scrapie cases were detected by tonsil biopsy at the next yearly sampling or at post mortem examination during the current year. As VRQ/ARR scrapie cases will be tonsil negative and only detected post-mortem, these cases may be incorporated relatively late in the computation of the incidence rate (in comparison of scrapie cases with tonsil positive biopsies).

### Mathematical modelling

We first describe the overall approach of the modelling, before we discuss the mathematical and statistical (i.e. parameter estimation) details.

#### Modelling approach

The main aim of our mathematical modelling is to quantify how the scrapie transmission risk in a flock is changing in time due to the selective breeding. The appropriate measure of the transmission risk is the basic reproduction number *R_0_*. It is defined in our context as the expected number of secondary cases of infection produced by a single primary scrapie infection in a population in which all animals of susceptible genotype are still uninfected [24-26]. *R_0 _*= 1 is the threshold value of the reproduction number below which the within-flock infection can not sustain itself and only small outbreaks can be expected in the flock [27,28]. The breeding programme can thus be considered successful if the value of *R_0 _*is brought below 1. After developing a model for scrapie transmission in a flock harbouring different genotypes, we can calculate the basic reproduction number as a function of the genotype frequencies in the flock. This enables us to evaluate the efficacy of the breeding programme in reducing the transmission risk by calculating, for each flock B to E individually, the time evolution of the basic reproduction number *R_0_*. For flocks A and F it was not possible to estimate *R_0_*, either because the number of scrapie cases was too low (flock A) or because the replacement strategy was so different from year to year that the sheep population did not have a stable age distribution (flock F). A stable host age distribution is a prerequisite for being able to define and calculate *R_0 _*for scrapie in sheep.

**Figure 1:**
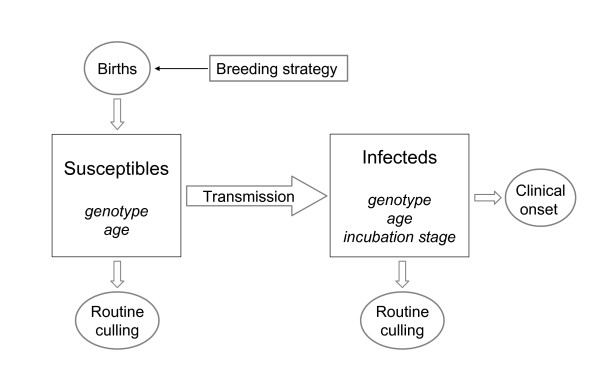
**Structure of our mathematical model of scrapie transmission in a sheep flock**.

In Figure 1 we schematically depict the different aspects to be taken into account in our scrapie transmission model. In our model, *R_0 _*at a given time *t *is expressed in terms of a number of estimable parameters. To calculate the effect of ram selection on *R_0_*, the following processes need to be taken into account in the model: population genetics of the flock, age-dependent culling and replacement of ewes, scrapie transmission and incubation. Therefore the estimable parameters include the age-genotype distribution at time *t*, the age-dependent survival functions of healthy and of infected sheep, and genotype-specific, age-dependent susceptibility parameters. These model parameters were all estimated from the observed data.

#### Modelling details

The calculation of *R_0 _*started by formulating a transmission model, designed as a tool to analyze how the transmission risk in a flock develops under selective breeding. This model describes the probability of a change in the infection status of an animal of age *a *and genotype *γ*, together with the probability of removal of the animal (i.e. fallen stock and animals sent for slaughter), in a way defined in Table 1 and Table 2. The age *a *runs from 0 (corresponding to the first year of life) until a flock-dependent maximum age. The model assumes lateral transmission between animals [29]. The role of the environment as a faecal-oral reservoir of infectivity is implicit in this assumption, as is the possible role of infectious placentas and transmission via milk [30]. These specific possible routes of transmission were left implicit in order to avoid introducing parameters that could not be estimated from the available data (e.g. it is as yet unclear how large the contribution to transmission of an infected placenta is, relative to other possible transmission routes). In a model explicitly including the placenta route the infectiousness of the placenta would need to be dependent on the genotype of the infected animal's fetus, in view the experimental results of Andreoletti et al. [31]. In our model the infectiousness of an infected animal is fully determined by its age and genotype; for the predicted effect of a breeding programme this is a conservative approximation. Another conservative model approximation made was that the infectiousness of infected animals is independent of the genotype. The relationship between infectiousness and incubation stage (or time since infection) was assumed to be linear, in close agreement with previous estimates of this dependence [6].

**Table 1:** Details of the transmission model

Status change of individuals:	Probability per unit of time:
Susceptible → infected	*g_γ_*(*a*)*λ* with 0 ≤ *g_γ_*(*a*) ≤ 1.
present → removed (non-infected animals)	$-\frac{1}{S\left(a\right)}\frac{dS}{da}\left(a\right),$
present → removed (infected animals)	$-\frac{1}{{\tilde{S}}_{\gamma }\left(a\right)}\frac{d{\tilde{S}}_{\gamma }}{da}\left(a\right)$

The symbols used are defined in Table 2.

**Table 2:** Definitions of model parameters and symbols. We estimated the model parameters *R_0_*(*0*), $S\left(a\right),{\tilde{S}}_{\gamma }\left(a\right)$ and *g_γ_*(*a*) for each flock individually

Symbol:	Interpretation:
*R_0_*(*0*)	The basic reproduction number in the flock just before the start of the breeding programme
*S*(*a*)	Survival function for non-infected animals
${\tilde{S}}_{\gamma }\left(a\right)$	Survival function for infected animals of genotype *γ*
*g_γ_*(*a*)	Relative susceptibility to infection of an animal of age *a *and genotype *γ*. It is determined by the parameters *p_γ _*and *α *below.
*β*(τ)	Level of infectiousness of an animal that acquired scrapie infection a time *τ *ago (*τ *= incubation stage of time since infection)
*f*_γ_(*t,a*)	Genotype distribution in the flock as a function of time
*R_0_*(*t*)	The basic reproduction number in the flock as a function of time. It is determined by all parameters listed in the rows above.
*p_γ_*	Probability of becoming infected in a certain year conditional on not having become infected before.
*α*	Age-dependency parameter
*λ*	Force of infection
*Q_γ_*(*a*)	The uninfected proportion of animals of age *a *and genotype *γ*

In Table 2 we defined the different variables and parameters of the model. The basic reproduction number *R_0_*(*t*) can be expressed in the following way in terms of *R_0_*(0) (i.e. its value at *t *= 0, just before the start of the breeding programme) and model parameters (as listed in Table 2):

(1) $$R_{\text{0}}\left(t\right)=R_{\text{0}}\left(0\right)\frac{\sum_{\gamma ,a}f_{\gamma }\left(t,a\right)S\left(a\right)g_{\gamma }\left(a\right)\sum_{\tau }\frac{{\tilde{S}}_{\gamma }\left(a+\tau \right)}{{\tilde{S}}_{\gamma }\left(a\right)}\beta \left(\tau \right)}{\sum_{\gamma }f_{\gamma }\left(0\right)\sum_{a}S\left(a\right)g_{\gamma }\left(a\right)\sum_{\tau }\frac{{\tilde{S}}_{\gamma }\left(a+\tau \right)}{{\tilde{S}}_{\gamma }\left(a\right)}\beta \left(\tau \right)}$$

In the above expression, *S *is the survival function for uninfected sheep, which was assumed to be independent of time (demographic equilibrium), and ${\tilde{S}}_{\gamma }$ is the survival function for infected animals of genotype *γ*. The function *f_γ_*(*t,a*) describes the genotype distribution in time, and was calculated directly from the genotyping data. *f_γ_*(0) is the genotype distribution at *t *= 0. The effect of the selective breeding programme was investigated by inspecting how *R_0 _*evolved in time as a result of the changes in *f_γ _*in time brought about by the breeding programme. We estimated the model parameters *R_0_*(0), $S\left(a\right),{\tilde{S}}_{\gamma }\left(a\right)$ and *g_γ_*(*a*) for each flock individually. The *τ *dependence of the infectiousness level was *β*(*τ*) taken to be linear, in close agreement with a sheep-to-sheep infectiousness profile estimated by Ferguson et al. [6]. We note that the estimated values for *R_0_*(*t*) are only weakly dependent on the shape of *β*(*τ*). This can be seen from Eq. (1), where *β*(*τ*) occurs in a similar way in both numerator and denominator. For a derivation of Equation 1 (for the special case of $\tilde{S}=S$, i.e. for the case that scrapie infection does not influence survival) we refer to Diekmann and Heesterbeek [32].

The calculation of *R_0_*(*t*) requires calculation of *R_0_*(0), i.e. the value of the basic reproduction number just before the start of the breeding programme. The approach we adopted for this calculation was tailored to an optimal use of the field data for flock C. For this flock the data were most informative due to the relatively large number of detected scrapie infections. The birth cohorts 1999 and 2000, that produced 20 cases out of the total of 31 detected in flock C, showed very similar incidence patterns: both the total scrapie incidence as well as the distribution of cases over age at first detection were almost the same for both cohorts. This motivated us to assume a constant force of infection for the time period in which the cases born in 1999 and 2000 were infected: λ(*t*) = λ = constant.

Clearly the force of infection should be ultimately reduced by the breeding programme if it is effective. However, due to the long incubation time of the infection, there will generally be a delay of a number of years between a reduction in *R_0 _*and the concomitant reduction in the force of infection. Because of this effect, the assumption of a constant force of infection as experienced by the 1999 and 2000 cohorts was a consistent approximation provided that most of the infections in these cohorts were occurring in the first few years of the animal's life.

For a constant force of infection, the basic reproduction number *R_0_*(0) just before the start of the breeding programme can be expressed in terms of the uninfected proportion *Q_γ_*(*a*) of animals of age *a *and genotype *γ *as follows:

(2) $$R_{\text{0}}\left(0\right)=\frac{\sum_{\gamma }f_{\gamma }\left(0\right)\sum_{a}S\left(a\right)g_{\gamma }\left(a\right)\sum_{\tau }\frac{{\tilde{S}}_{\gamma }\left(a+\tau \right)}{{\tilde{S}}_{\gamma }\left(a\right)}\beta \left(\tau \right)}{\sum_{\gamma }f_{\gamma }\left(0\right)\sum_{a}Q_{\gamma }\left(a\right)S\left(a\right)g_{\gamma }\left(a\right)\sum_{\tau }\frac{{\tilde{S}}_{\gamma }\left(a+\tau \right)}{{\tilde{S}}_{\gamma }\left(a\right)}\beta \left(\tau \right)}.$$

#### Parameter estimation

As the non-infected proportions *Q_γ_*(*a*) can not be estimated directly from the field data (because "negative" is not the same as "non-infected"), we relate these to a number of model parameters which subsequently will be estimated from the field data. We express the proportions *Q_γ_*(*a*) in terms of the conditional probability *p_γ_*(*a*) of being infected at age *a *(for details we refer to the Additional file 1). The proportions *Q_γ_*(*a*) can then be expressed in terms of a (small) number of parameters by choosing model parameterizations for *p_γ_*(*a*). The simplest model (containing only one parameter) is an age-independent probability:

(3) $$Model\;I:p_{\gamma }(a)=p_{\gamma }.$$

Slightly more complicated models (containing two parameters) are the following: *p_γ_*(*a*) decreases exponentially with age:

(4) $$Model\;II:p_{\gamma }\left(a\right)=p_{\gamma }\alpha^{a},\text{ with }0\leq \alpha \leq 1;$$

or *p_γ_*(*a*) is reduced only after the first year of life and remains the same afterwards:

(5) $$Model\;III:p_{\gamma }(0)=p_{\gamma },\text{ and }p_{\gamma }\left(a\right)=\alpha p_{\gamma }\;\text{for }a>0,\text{ with }0\leq \alpha \leq 1.$$

We assumed that infections of ARQ/ARQ animals born in 1999 or 2000 in flock C will become first detected at most two years after the end of the year of life in which the infection took place. This assumption was motivated by the fact that 19 out of 20 cases detected in flock C were three or less years old when first detected. Based on this assumption, the delay between infection and detection could be described using two estimable parameters defined in the Additional file 1. Including also the parameters *p*_γ = ARQ/ARQ _(all Models) and *a *(Models II and III) we thus worked with 3 (Model I) or 4 (Models II and III) estimable parameters. The model likelihood (given in the Additional file 1) was numerically optimized to obtain maximum-likelihood estimates for these parameters. For each of the models, the model fit was judged by Pearson's Chi-square statistic (and associated *P*-value from a chi-square approximation).

As for the genotype ARQ/ARR in flock C there were no cases observed, it was not possible to apply the above approach of analysis to this genotype (although the point estimate of *p*_γ = ARQ/ARR _was easily calculated (*p*_γ = ARQ/ARR _= 0), we would also seek to calculate an upper confidence bound of *p*_γ = ARQ/ARR_). Furthermore, for all genotypes in flocks B, D and E the number of scrapie cases observed is too limited to apply the above analysis. For these flocks/genotypes we therefore followed a more pragmatic approach, in which we derived a pessimistic estimate of *R_0_*(0) by using an estimate of the non-infected proportions *Q_γ_*(*a*) based on the cohort with the highest incidence in the data. These cohorts are in all cases "early" cohorts, i.e. from before the start of the breeding programme. For this cohort we calculated the observed total fraction positive F*_γ_*^+ ^as the number of detected scrapie cases divided by the total number of animals of genotype *γ *which were either found test positive or at least reached a given minimum age without being found positive (to avoid misclassifying animals that were infected but not yet incubating long enough to be detected). For further details we refer to the Additional file 1. We note that as the model calculations are not using the scrapie infection data for "late" cohorts (as they only use data for early cohorts to estimate the starting value *R_0_*(0)), they are not affected by any right-censoring problems of detecting scrapie in the late cohorts.

## Results

### Genotype distribution

**Figure 2:**
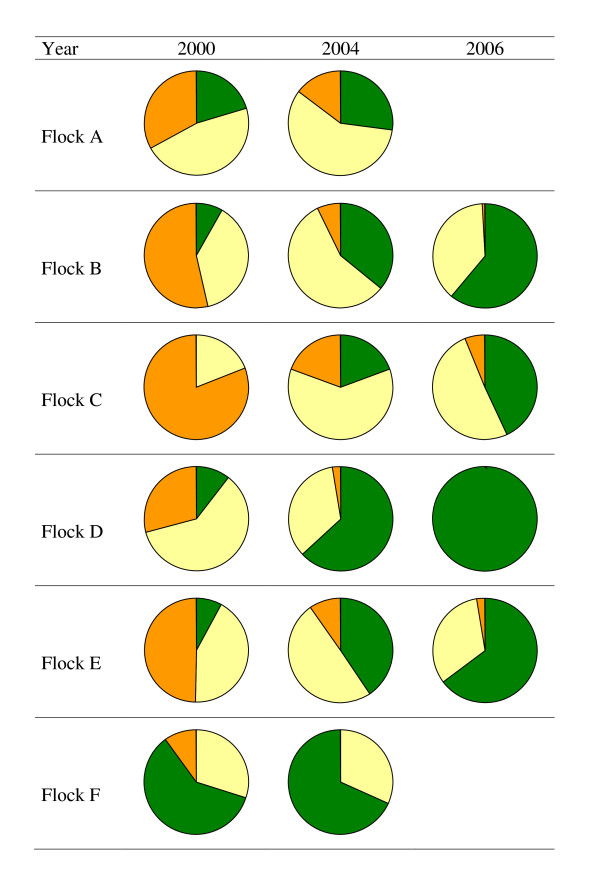
**Distribution of the genotypes ARR/ARR (green), S/ARR (yellow) and S/S (orange) during the study period (four years for flock A & F; six years for flock B, C, D & E)**. Alleles different from the ARR allele are denoted by S (of susceptibility); in the present case this means that S = ARQ, AHQ or VRQ.

To monitor the genotype distribution, the results of in total 1175 sheep were used. Four alleles (VRQ, ARQ, AHQ and ARR) were observed in this study, and each one of the corresponding ten possible genotypes occurred in the study flocks. Pie charts in Figure 2 show how the observed genotype distribution of the ewe population in each of the six flocks evolved during the study period. In flocks A-E we observed a genetic improvement by an increase of the ARR allele and the ARR/ARR genotype in these flocks, with even an increase up to 100% for flock D in 2006. Although in flock F ARR/ARR rams were used, unfortunately the farmer did not use the offspring of these rams to replace ewes. As a result hardly any genetic improvement was achieved. Monitoring of flock F was ceased late 2004 after the flock was sold by the owner.

More details on the genotype distribution of each flock can be found in Table S1 of the Additional file 1.

### Scrapie cases

To monitor scrapie infections 3819 tonsil biopsies and post-mortem samples (tonsils and obex) of 1168 sheep were analysed for the presence of PrP^Sc^. Eleven percent of the tonsil biopsy samples were considered unsuited for diagnosis as the tonsil biopsy contained <3 lymphoid follicles. Six of the 1168 sheep (0.5%) were unsuited due to severe autolysis. For 44 post-mortem sheep (3.8%) the tonsils were not available and for 3 sheep (0.3%) the obex was missing due to inadequate sampling.

In total 59 scrapie cases were detected during the entire monitoring period, of which 51 cases were sheep without the ARR allele (2 VRQ/VRQ, 16 VRQ/ARQ, 33 ARQ/ARQ) and 8 cases were VRQ/ARR (Table 3). No scrapie cases were detected in birth cohorts from 2001 onwards in any of the flocks, i.e. in animals born after the start of the control programme in 2000. The eight VRQ/ARR cases were only detected by positive obex during post-mortem analysis, with all tonsil biopsies of these animals as well as the tonsils collected at post mortem being negative. For 36 of the 51 cases without the ARR allele, scrapie was detected first by tonsil biopsy when alive, and later confirmed by positive post-mortem analysis. For most of the 15 remaining cases recent tonsil biopsy data were missing for different reasons. Detailed information on the 59 scrapie can be found in Table S2 of the Additional file 1.

**Table 3:** Total number of scrapie cases found in sheep of different genotype during the monitoring period

	Number of scrapie cases of genotype:					
	
Flock	VRQ/VRQ	VRQ/ARQ	VRQ/ARR	ARQ/ARQ	ARQ/ARR	total
A	-	1	-	-	-	1
B	-	5	-	1	-	6
C	-	-	-	31	-	31
D	-	6	3	-	-	9
E	1	3	-	1	-	5
F	1	1	5	-	-	7

	2	16	8	33	0	59

Flocks B-F were affected by a substantial scrapie infection (Table 3). Flock C is the most heavily affected flock, in which in total 31 scrapie cases were detected during the monitoring period, all of ARQ/ARQ genotype. Given the high incidence in this flock despite the absence of the VRQ allele, one might hypothesize that this outbreak involved a scrapie strain different from the strain(s) affecting the other flocks. Unfortunately the remaining flock A could not provide information on the effect of the breeding programme on scrapie transmission, as the scrapie outbreak in this flock faded out one year after the selective breeding programme started. Only a single scrapie case was detected in 2000 by tonsil biopsy and this sheep was culled in 2001. Up until (and inclusive) the last biopsy round in 2004 no further positives were found. Therefore, monitoring of flock A was ceased after that year.

Table 4 and Table 5 show the prevalence and incidence rate of scrapie in the six flocks throughout the monitoring period, irrespective of genotype. A decline of scrapie cases in time was found in the four flocks B-E. This decline in both scrapie prevalence and incidence provides direct evidence for the efficacy of the breeding programme. Furthermore, none of the animals born after the start of the programme was found scrapie positive. Given the prevalence data shown in Table 4, it is unlikely (flock B) or even very unlikely (flocks C-E) that a stochastic extinction of scrapie would have resulted in any of these flocks, had selective breeding not been applied. Further evidence for the efficacy of the breeding programme is given by the results for the basic reproduction number presented below. Flock F did not comply with the breeding programme (see next section); here a low scrapie prevalence remained throughout the four years of monitoring.

**Table 4:** Prevalence of scrapie (in %) based on tonsil biopsy* collected at yearly samplings of live animals

Year	Flock					
	
	A	B	C	D	E	F
2000	0.8 (1/121)	1.9 (2/108)	7.3 (8/109)	10.4 (5/48)	2.7 (4/149)	6.9 (2/30)
2001	0.0 (0/107)	1.9 (2/105)	6.4 (7/109)	7.3 (3/41)	2.5 (4/159)	0.0 (0/37)
2002	0.0 (0/115)	0.9 (1/108)	8.9 (9/101)	3.2 (1/31)	0.6 (1/154)	0.0 (0/46)
2003	0.0 (0/107)	1.0 (1/105)	4.7 (5/107)	0.0 (0/29)	0.6 (1/155)	5.0 (1/20)
2004	0.0 (0/102)	0.0 (0/109)	0.0 (0/123)	0.0 (0/38)	0.0 (0/175)	5.3 (1/19)
2005	n.d.**	0.0 (0/115)	0.0 (0/144)	0.0 (0/47)	0.0 (0/205)	n.d.
2006	n.d.	0.0 (0/110)	0.0 (0/155)	0.0 (0/23)	0.0 (0/234)	n.d.

Between brackets: # positive samples/# tested ewes

**Table 5:** Incidence rates based on newly detected scrapie cases (by tonsil biopsy or by post mortem examination) divided by the number of sheep-years at risk

Period	Flock					
	
	A	B	C	D	E	F
2000-2001	0.00 (0)	0.02 (2)	0.07 (7)	0.02 (1)	0.00 (0)	0.00 (0)
2001-2002	0.00 (0)	0.02 (2)	0.06 (6)	0.00 (0)	0.01 (1)	0.02 (1)
2002-2003	0.00 (0)	0.00 (0)	0.09 (8)	0.09 (3)	0.00 (0)	0.08 (3)
2003-2004	0.00 (0)	0.00 (0)	0.01 (1)	0.00 (0	0.00 (0)	0.05 (1)
2004-2005	n.d.**	0.00 (0)	0.01 (1)	0.00 (0)	0.00 (0)	n.d.
2005-2006	n.d.	0.00	0.01 (1)	0.00 (0)	0.00 (0)	n.d.

*if available also post mortem results

**n.d. = not done

Between brackets: # newly detected scrapie cases

### Parameter estimates

The main parameter of interest in our analysis is *R_0_*(*t*), and the other parameters are mainly relevant as determinants of *R_0_*(*t*) (through Eq.(1)). However, the results of the parameter estimation for the Models I-III (introduced in section 2.4.3) are also interesting by themselves as they describe possible age-dependence of transmission. Table 6 displays these estimates based on the data for flock C. The best fit was obtained for Model III, as this model had the lowest value for the Chi-square statistic and the highest *P *value (*P *= 0.38). The maximum-likelihood estimates for the two parameters describing the age-dependent infection probability (see Eq. (5)) are: *p*_ARQ/ARQ _= 0.416 (0.273, 0.574) and α = 0.059 (0.003, 0.29). The 95% confidence intervals (in parentheses) were constructed with the score test [33]. The score test was used (and not the likelihood ratio test) to avoid any problems with the chi-square approximation of the likelihood ratio test for parameter values close or equal to the domain boundaries 0 and 1. We consider the fit of model III to be quite satisfactory, given that the data (see Additional file 1: Table S4) comprised an exceptional animal that was first found positive at the old age of six years. When this late positive was removed from the dataset, both Models II and III yielded perfect fits (*P *= 1). We note that the age-dependency parameter *α *in Model III is significantly smaller than 1 (upper confidence bound equals 0.29). Therefore, Model III with α = 1 (age-dependent susceptibility), is in fact rejected.

**Table 6:** Comparison of the quality of the model fit for different model alternatives

Description of model variant	Number of free parameters	Pearson's Chi-square statistic	*P *value for Goodness of fit
(i) Model I (Eq. (3))	3	8.9	0.06
(ii) Model II (Eq. (4))	4	8.1	0.09
(iii) Model III (Eq. (5))	4	3.1	0.38

For the parameter estimation results for flock B, D and E and for *p*_γ = ARQ/ARR _in flock C we refer to the Additional file 1, as their main relevance is in determining the *R_0_*(*t*) estimates presented in the next section.

### Effect of the breeding programme on scrapie transmission

**Figure 3:**
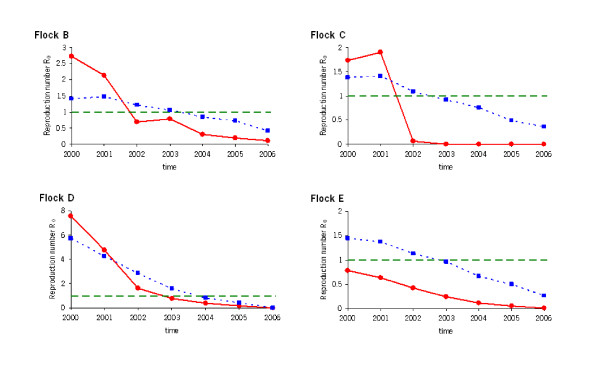
**Change in time of the basic reproduction number *R_0_*(*t*) for flock B-E**. Circles represent *R_0 _*values calculated using point estimates for the relative susceptibility *g_γ _*of different genotypes and age classes. Squares represent *R_0 _*values calculated using upper-bound estimates for the relative susceptibility of S/ARR genotypes and lower-bound estimates for the relative susceptibility of S/S genotypes and using an age-independent susceptibility model (*α *= 1). This combination of parameter choices yields the most conservative prediction of the effect of the breeding programme, i.e. the squares serve as upper confidence bounds when they exceed the results depicted as circles.

For each of the four flocks B-E we calculated *R_0 _*as a function of time (Figure 3). For flocks A and F it was not possible to estimate *R_0 _*for reasons explained in section 2.4.1. The squares display *R_0 _*calculated using upper-bound estimates for the susceptibility of S/ARR genotypes and lower-bound estimates for the susceptibility of S/S genotypes, and further assuming that scrapie susceptibility is age independent in all four flocks. This combination of parameter choices yields the most conservative model prediction of the effect of the breeding programme. The corresponding *R_0 _*results (squares in Figure 3) therefore serve as upper confidence bounds when they exceed the point estimate results (circles).

The point estimates of *R_0 _*are obtained from point estimates of the age-dependent, genotype-specific susceptibility parameters in the model. From Figure 3 we thus conclude that in flocks B, D and E *R_0 _*is reduced to values significantly below one after four years, and in flock C this is the case already after three years. Figure 3 shows that for all three flocks B-D the point estimates of *R_0_*(circles) are reduced below the threshold value *R_0 _*= 1 within at most three years. For flock E the point estimate of *R_0 _*is already below threshold at the start of the study period, however the upper confidence bound is not. It is relevant to note that the results for flocks B, D and E illustrate that scrapie control (i.e. when the upper confidence bound for *R_0 _*declines to below 1) can be achieved before the presence of non-ARR/ARR animals has reduced to zero. This can be seen from the *R_0 _*point estimates, which are non-zero implicating the presence of non-ARR/ARR animals.

## Discussion

Six commercially-run flocks were selected to evaluate the effect of a breeding programme for scrapie control, consisting of selecting homozygote resistant rams for mating. We used genotyping to monitor the genotype distribution, and tonsil biopsies and post-mortem analyses to monitor the occurrence of scrapie infection throughout the study period of six years. The results show that the breeding program leads to rapid outbreak control, typically within at most four years of selective breeding. Furthermore, our results show that to reach the situation where *R_0 _*< 1 (i.e. outbreak control) the number of non-ARR/ARR animals does not have to be reduced to zero in the flock.

For the four flocks of interest, B, C, D and E, we used mathematical modelling to estimate the basic reproduction number *R_0 _*through time, separately for each flock. This approach of combining observational data with modelling is important to obtain more insight in the dynamics of the infection, in particular for slow-progressing diseases like scrapie. Right-censoring of scrapie detection in the late birth cohorts of sheep may have resulted in a slight underestimation of the prevalence and incidence of the latest years of the monitoring period. However, the possible right-censoring did not affect the calculation of *R_0_*, as this calculation is only using the scrapie infection data of early cohorts. At the start of the programme, the four within-flock *R_0 _*values were estimated to be in the range of 0.8-8.0. This range is of the same order of magnitude as previous estimates of the within-flock *R_0 _*for other scrapie outbreaks by Matthews et al. [34] (3.9) and Hagenaars et al. [35] (2-7.5).

Our results show that to reach the situation where *R_0 _*becomes significantly smaller than 1 (as requested for outbreak control) the number of non-ARR/ARR animals does not have to be reduced to zero in the flock. On a national scale this implies that it will be possible to maintain the diversity of susceptibility alleles desired in case a new scrapie (or TSE) strain targeting ARR would arise in the future. It is important to note that when a reduction of *R_0 _*to below one is achieved, this does not mean that the breeding programme can simply be ceased. Although expected to decline with time, infection risks to susceptible genotypes, e.g. from a scrapie-contaminated environment, are expected to remain present for some time. Therefore, re-introducing the use of non-ARR/ARR rams for breeding without risking to revert to a situation with *R_0 _*> 1 requires a new breeding strategy that avoids mating such rams with non-ARR/ARR ewes.

An intermediate modelling result of interest relates to flock C. The relatively high number of scrapie cases observed in this flock allowed a detailed estimation of a model parameter controlling age-dependency of scrapie susceptibility. It was found that in this flock, the null hypothesis of an age-independent susceptibility had to be rejected. The maximum-likelihood estimate of the age-dependency parameter corresponds to a scenario in which scrapie infection is occurring predominantly in the first year of life. This is in line with what is often assumed to be the case, at least in flocks affected by substantial scrapie outbreaks, but has only rarely been established based on quantitative evidence [36].

Flocks B to F were affected by a substantial scrapie infection at the start of the breeding programme, and except for flock C scrapie was mainly associated with the VRQ allele. Flock C was the most heavily affected flock, in which in total 31 scrapie cases were detected, all of ARQ/ARQ genotype. Given the high incidence in this flock despite the absence of the VRQ allele in the sheep population, one might hypothesize that this outbreak involved a scrapie strain different from the strain(s) affecting the other flocks. Certainly, in this flock the estimated (relative) susceptibility of the ARQ/ARQ genotype was much higher than in the other flocks.

We expect that possible between-strain differences in genetic susceptibility will not limit the effectiveness of the breeding strategy found here, given that no classical scrapie strain thus far has escaped ARR-associated resistance. In line with this expectation, the breeding programme proved successful in all four flocks analyzed despite between-flock differences in the estimated genotype-specific susceptibilities.

As a result of the control programme not only the reproduction number *R_0_*, but also the infection pressure (or force of infection) in the field will decrease in time. However, due to the long incubation period of a scrapie infection, a delay of a few years is expected between the reduction in *R_0 _*and the reduction in infection pressure. This is in line with the detection of new scrapie cases during this monitoring study, which were all born before the start of the programme. Extending the breeding strategy by a removal of scrapie-susceptible ewes on the basis of their genotype would accelerate the reduction of both *R_0 _*as well as the infection pressure. However, when considering the rapid outbreak control as observed in this study, the use of resistant rams seems sufficient and can be recommended as a control strategy in scrapie-affected countries.

## Competing interests

The authors declare that they have no competing interests.

## Authors' contributions

GN, HvR, and PV participated in the design of the study; GN, TH and LvK performed research; GN, TH and BE analyzed data; GN, HvR, and TH drafted the manuscript. All authors read and approved the final manuscript.

## Supplementary Material

Additional file 1 **Supplementary data**. Tables with details of genotype distribution of the six flocks and detailed information of all scrapie cases are shown. Details of quantitative analyses are provided: the calculation of the non-infected proportions Q_γ _(a), the estimation of parameters for γ = ARQ/ARQ in flock C, the estimation of parameters for flock B, D and E and of upper confidence bound for p_ARQ/ARR _in flock C, the calculation of upper confidence bound for R_0_, and the distribution of survival times. Click here for file
