# Supplementary material for: Breeding with resistant rams leads to rapid control of classical scrapie in affected sheep flocks
Source: Vet Res. 2011 Jan 11;42(1):5. doi: 10.1186/1297-9716-42-5 (PMC3037897; doi:10.1186/1297-9716-42-5)
Supplement: Additional file 1 — Supplementary data. Tables with details of genotype distribution of the six flocks and detailed information of all scrapie cases are shown. Details of quantitative analyses are provided: the calculation of the non-infected proportions Qγ (a), the estimation of parameters for γ = ARQ/ARQ in flock C, the estimation of parameters for flock B, D and E and of upper confidence bound for pARQ/ARR in flock C, the calculation of upper confidence bound for R0, and the distribution of survival times. [file 1297-9716-42-5-S1.DOC]

### Additional file 1

To: “Breeding with resistant rams leads to rapid control of classical scrapie in affected sheep flocks” by Gonnie Nodelijk et al.

**Genotype distribution**

Details of the genotype distribution of the six flocks are shown in Table S1.

**Scrapie cases**

Detailed information of the 59 scrapie cases is shown in Table S2.

**Mathematical modelling and calculation of *R_0_***

**Calculation of the non-infected proportions Q_γ_ (a)**

As the non-infected proportions *Q_γ_* (*a*) can not be estimated directly from the field data (because “negative” is not the same as “non-infected”), we relate these to a number of model parameters which subsequently will be estimated from the field data. When assuming a Poisson process for the number of infectious contacts, the conditional probability $p_{\gamma}\left(a\right)=p_{\gamma}(x=a|x>a-1)$ of being infected at age *x = a* equals the probability that at least one infectious contact takes place during the *a*-th year of age:

$p_{\gamma}(a)=1-e^{-\lambda g_{\gamma}\left(a\right)}$.

For the definitions of $g_{\gamma}\left(a\right)$ and $\lambda$we refer to Table 2 in the main text. The non-infected proportions *Q_γ_* (*a*) of animals of age *a* and genotype *γ* can be expressed in terms of the probabilities $p_{\gamma}\left(a\right)$ as follows:

$Q_{\gamma}(a)=\prod_{i=0}^{a-1} (1-p_{\gamma}\left(i\right))$.

The proportions *Q_γ_* (*a*) can now be expressed in terms of a (small) number of parameters by choosing model parameterizations for$p_{\gamma}\left(a\right)$ as explained in the main text (Eq. 3-5).

**Parameter estimation**

*Estimation of parameters for γ=ARQ/ARQ in flock C*

For the case of flock C we estimated the model parameters for animals of genotype ARQ/ARQ from the field data by Maximum-Likelihood estimation, as follows. The data for flock C were grouped together in 7 cells, for *a*= 1 until *a*= 7, where each cell represents the realization of the chance process described by the conditional probability$P(x=a|x>a-1)$that an arbitrary animal with genotype ARQ/ARQ and age *a* will be found positive for the first time, given that the animal was not yet found positive until age $a-1$. Such a realization was given by two integer numbers, *n*(*a*) and *m*(*a*), where *n*(*a*) was the number of animals still present at age *a* and not yet found positive until age $a-1$, and *m*(a) the number of animals from this that was found positive for the first time before or around reaching age *a*. By combination of the data for the cohorts 1999 and 2000 we obtained the realizations as shown in Table S3.

The conditional probability$P(x=a|x>a-1)$can be written as follows:

$$P(x=a|x>a-1)=\frac{P(x=a)}{P(x>a-1)}=\frac{P(x=a)}{1-\sum_{x=1}^{a-1} P(x)}.$$

Here *x* was the age at which the animal is found positive for the first time, either by a positive tonsil biopsy or by a positive post mortem test. $P(x=a)$ was the probability for an arbitrary animal of genotype ARQ/ARQ to be found positive at age *a* for the first time. We introduced the parameters *h_i_* that described the delay between infection and detection. *h*_1_ is the probability that an animal first becomes test-positive at the end of the year of age in which it was infected, *h*_2_ is the probability that this is the case one year later, and *h*_3_ the probability that this is the case two years later. It followed that:

$P(x=a)=p\left(0\right)h_{a}+\sum_{i=1}^{a-1} p\left(i\right)h_{a-i}\prod_{k=0}^{i-1} (1-p\left(k\right))$.

Here $p\left(i\right)=p_{\gamma=\text{ARQ/ARQ}}\left(i\right)$.

We assumed that infections of ARQ/ARQ animals born in 1999 or 2000 in flock C will become first detected at most two years after the end of the year of life in which the infection took place; i.e. the three 3 *h*-parameters summed up to 1. This assumption was motivated by the fact that 19 out of 20 cases detected were three or less years old when first detected. Next to the parameters *p_γ =_*_ARQ/ARQ_ and *α* we thus introduced two free *h*-parameters. The likelihood (*L*) can be expressed in terms of the conditional probabilities $P\left(x=a|x>a-1\right)$ in the following product-binomial form:

$$L=\prod_{a=1}^{7} \left(\begin{matrix} n(a) \\ m(a) \end{matrix}\right)\left(P\left(x=a|x>a-1\right)\right)^{m\left(a\right)}\left(1-P\left(x=a|x>a-1\right)\right)^{n\left(a\right)-m\left(a\right)}.$$

For every combination of the four model parameters the conditional probabilities can be calculated and from these the likelihood.

*Estimation of parameters for flock B, D and E and of upper confidence bound for p_ARQ/ARR_ in flock C*

For these flocks/genotypes we derived a pessimistic estimate of *R_0_*(0) by using an estimate of the non-infected proportions *Q_γ_* (*a*) based on the cohort with the highest incidence in the data. For this cohort we calculated the observed total fraction positive F*_γ_*^+^ as the number of detected scrapie cases divided by the total number of animals of genotype *γ* which were either found test positive or at least reached a given minimum age without being found positive. For flock B this minimum age was set equal to two years (all cases in the relevant cohort in this flock were detected by two years of age), for the other flocks it is four years. If for particular genotypes in a flock there was no information at all in the data for the highest-incidence cohort (no positives and no negatives reaching three or more years of age), we borrowed relative susceptibility information for this genotype from other flocks.

We made the conservative assumption of an age-independent susceptibility (*α *= 1). Also we assumed that the observed total fraction positive F*_γ_*^+^ in the highest-incidence cohorts in flocks D and E was entirely due to infections arising in the first two years of life, arising from a constant endemic infection probability *p_γ_*. This means that we assumed that infections caused in later years of life, if any, were too recent to be detected by the age of four years, which was a conservative assumption too. In contrast, and this was the only approximation that was not conservative, we assumed that the infections arising in the first two years of life were all detected by the time the animals reach the age of four years: $F_{\gamma}^{+}=p_{\gamma}+p_{\gamma}\left(1-p_{\gamma}\right).$ In flock B, to account for the lower minimum age used in calculating the total fraction positive, we used the approximation $F_{\gamma}^{+}=p_{\gamma}.$ The results for $p_{\gamma}$ are listed in Table S4. To obtain an indication of quality of the calculation of $R_{0}\left(t\right)$ used for flocks B, D and E, we applied the same calculational approach to flock C in order to compare the results with those of the more sophisticated analysis for flock C. This comparison is shown in Figure S1. The upper confidence bound curves of $R_{0}\left(t\right)$ of the two analyses are very similar and the curves drop below 1 at almost the same moment in time. Also the point estimate curves drop below 1 at almost the same moment, however with the result of the more sophisticated analysis declining much sharper. The latter difference arises mainly because the more sophisticated analysis uses the estimated age-dependent susceptibility ($\alpha=0.059$) whereas the other analysis assumes an age-independent susceptibility ($\alpha=1$).

*Calculation of upper confidence bound for R_0_*

The breeding programme replaces S/S animals with more resistant ones. To estimate the upper confidence bound of *R_0_* we therefore used the combination of *p_γ_* confidence bounds for which the gain in resistance (associated with replacing S/S animals with more resistant genotypes) is minimal. Therefore we used the lower confidence limits for the *p_γ_*’s with *γ  *= S/S and the upper confidence limits for the other genotypes, requiring in addition that the susceptibility of animals of homozygote susceptible genotype was not lower than that of animals of heterozygote ARR genotype, and that the genotype VRQ/X was at least as susceptible as ARQ/X.

We note that using this recipe, the parameter confidence bound results for flock B in Table SIV in fact lead us to the maximally conservative assumption, when calculating of the upper confidence bound of *R_0_* in flock B, that the four genotypes VRQ/ARQ, ARQ/ARQ, VRQ/ARR and ARQ/ARR are equally susceptible. We set the upper confidence bound for *p*_ARQ/ARR_ in flock D equal to one-tenth of that of VRQ/ARR animals based on estimates by Baylis et al. [1]. Our results for *R_0_* were essentially insensitive to the value of *p_γ_*_= VRQ/VRQ_, which we had set equal to 1 throughout.

**Distribution of survival times**

The distribution of survival times (survival function) $S$of sheep in a flock determines the age distribution in demographic equilibrium, and for that reason $S$ appears in the expression for *R_0_*. The survival curve of infected animals $\tilde{S}_{\gamma}$ also appears in the expression of *R_0_*, as it determines how long infected animals can contribute to the infection pressure. Both these distributions were estimated from the data. For each of the flocks separately we estimated the overall survival curve $S$ for non-scrapie ewes (irrespective of genotype). The results are shown in Figure S2. We also estimated the survival curve for the detected scrapie cases, and compared it to $S$**.** In Figure S3 the survival function of the non-scrapie ewes was compared to that of the detected scrapie cases for flock C. In this Figure we also included simple parametric curves fitted to the observed curves. As these cases were all of genotype ARQ/ARQ, the detected-scrapie survival curve is a measure of $\tilde{S}_{ARQ/ARQ}$.

No significant difference has been found between the survival function of non-scrapie ewes and that of scrapie ewes in either of the flocks B, D, E and F (not shown). In these flocks were therefore assumed that $\tilde{S}_{\gamma}=S$.

**References**

[1] Baylis M, Chihota C, Stevenson E, Goldmann W, Smith A, Sivam K, Tongue S, Gravenor MB**: Risk of scrapie in British sheep of different prion protein genoty**pe*. J Gen Vir*ol 2004**,** 85:2735-2740.

**Figure S1.** Comparison of results for $\text{R}_{0}\left(t\right)$ in flock C when using the approach used for flocks B, D and E (full lines), with the results of the more sophisticated analysis for flock C (dashed lines). Upper panel, red lines: point estimates. Lower panel, blue lines: Upper confidence bounds.

**Figure S2.** Survival curves of non-scrapie ewes of flocks B−E.

**Figure S3.** Survival curves of ewes of flock C.

**Table S1. PrP genotypes distribution in six sheep flocks during the study period (four years for flock A and F and six years for flock B-E).**

|  | Flock A: # ewes (%) per year | | | | |
| --- | --- | --- | --- | --- | --- |
| Genotype | 2000 | 2001 | 2002 | 2003 | 2004 |
| ARR/ARR | 25 (20) | 18 (17) | 17 (15) | 21 (20) | 27 (26) |
| AHQ/ARR | 1 (1) | 0 (0) | 0 (0) | 0 (0) | 0 (0) |
| AHQ/AHQ | 0 (0) | 0 (0) | 0 (0) | 0 (0) | 0 (0) |
| ARQ/ARR | 48 (40) | 54 (50) | 63 (55) | 61 (57) | 56 (55) |
| ARQ/AHQ | 2 (2) | 0 (0) | 0 (0) | 0 (0) | 0 (0) |
| ARQ/ARQ | 33 (27) | 28 (26) | 28 (24) | 21 (19) | 15 (15) |
| VRQ/ARR | 7 (6) | 5 (5) | 7 (6) | 3 (3) | 3 (3) |
| VRQ/AHQ | 0 (0) | 0 (0) | 0 (0) | 0 (0) | 0 (0) |
| VRQ/ARQ | 5 (4) | 2 (2) | 0 (0) | 0 (0) | 0 (0) |
| VRQ/VRQ | 0 (0) | 0 (0) | 0 (0) | 0 (0) | 0 (0) |
| Missing | 0 (0) | 0 (0) | 0 (0) | 1 (1) | 1 (1) |
| Total | 121 (100) | 107 (100) | 115 (100) | 107 (100) | 102 (100) |

|  | Flock B: # ewes (%) per year | | | | | | |
| --- | --- | --- | --- | --- | --- | --- | --- |
| Genotype | 2000 | 2001 | 2002 | 2003 | 2004 | 2005 | 2006 |
| ARR/ARR | 9 (8) | 8 (8) | 17 (16) | 27 (26) | 38 (35) | 50 (43) | 67 (61) |
| AHQ/ARR | 1 (1) | 2 (2) | 2 (2) | 3 (3) | 2 (2) | 0 (0) | 0 (0) |
| AHQ/AHQ | 1 (1) | 0 (0) | 0 (0) | 0 (0) | 0 (0) | 0 (0) | 0 (0) |
| ARQ/ARR | 34 (31) | 34 (32) | 44 (40) | 54 (51) | 53 (49) | 53 (46) | 37 (33) |
| ARQ/AHQ | 4 (4) | 7 (7) | 4 (4) | 1 (1) | 1 (1) | 1 (1) | 0 (0) |
| ARQ/ARQ | 41 (38) | 40 (38) | 31 (29) | 8 (7) | 6 (5) | 2 (2) | 1 (1) |
| VRQ/ARR | 6 (6) | 4 (4) | 4 (4) | 6 (6) | 5 (5) | 5 (4) | 4 (4) |
| VRQ/AHQ | 0 (0) | 0 (0) | 0 (0) | 0 (0) | 0 (0) | 0 (0) | 0 (0) |
| VRQ/ARQ | 12 (11) | 10 (9) | 5 (4) | 5 (5) | 1 (1) | 1 (1) | 0 (0) |
| VRQ/VRQ | 0 (0) | 0 (0) | 0 (0) | 0 (0) | 0 (0) | 0 (0) | 0 (0) |
| Missing | 0 (0) | 0 (0) | 1 (1) | 1 (1) | 3 (2) | 3 (3) | 1 (1) |
| Total | 108 (100) | 105 (100) | 108 (100) | 105 (100) | 109 (100) | 115 (100) | 110 (100) |

|  | Flock C: # ewes (%) per year | | | | | | |
| --- | --- | --- | --- | --- | --- | --- | --- |
| Genotype | 2000 | 2001 | 2002 | 2003 | 2004 | 2005 | 2006 |
| ARR/ARR | 0 (0) | 0 (0) | 1 (1) | 7 (7) | 23 (19) | 46 (32) | 67 (43) |
| AHQ/ARR | 0 (0) | 0 (0) | 0 (0) | 0 (0) | 3 (2) | 3 (2) | 3 (2) |
| AHQ/AHQ | 0 (0) | 0 (0) | 0 (0) | 0 (0) | 0 (0) | 0 (0) | 0 (0) |
| ARQ/ARR | 21 (19) | 21 (19) | 32 (32) | 60 (56) | 71 (58) | 78 (54) | 74 (48) |
| ARQ/AHQ | 5 (5) | 5 (5) | 2 (2) | 2 (2) | 2 (1) | 2 (1) | 1 (1) |
| ARQ/ARQ | 83 (76) | 83 (76) | 66 (65) | 38 (35) | 22 (18) | 14 (10) | 8 (5) |
| VRQ/ARR | 0 (0) | 0 (0) | 0 (0) | 0 (0) | 0 (0) | 0 (0) | 0 (0) |
| VRQ/AHQ | 0 (0) | 0 (0) | 0 (0) | 0 (0) | 0 (0) | 0 (0) | 0 (0) |
| VRQ/ARQ | 0 (0) | 0 (0) | 0 (0) | 0 (0) | 0 (0) | 0 (0) | 0 (0) |
| VRQ/VRQ | 0 (0) | 0 (0) | 0 (0) | 0 (0) | 0 (0) | 0 (0) | 0 (0) |
| Missing | 0 (0) | 0 (0) | 0 (0) | 0 (0) | 2 (2) | 1 (1) | 2 (1) |
| Total | 109 (100) | 109 (100) | 101 (100) | 107 (100) | 123 (100) | 144 (100) | 155 (100) |

|  | Flock D: # ewes (%) per year | | | | | | |
| --- | --- | --- | --- | --- | --- | --- | --- |
| Genotype | 2000 | 2001 | 2002 | 2003 | 2004 | 2005 | 2006 |
| ARR/ARR | 5 (10) | 7 (17) | 10 (32) | 15 (52) | 24 (63) | 32 (68) | 23 (100) |
| AHQ/ARR | 5 (10) | 4 (10) | 5 (16) | 4 (14) | 5 (13) | 5 (11) | 0 (0) |
| AHQ/AHQ | 0 (0) | 0 (0) | 0 (0) | 0 (0) | 0 (0) | 0 (0) | 0 (0) |
| ARQ/ARR | 12 (25) | 11 (27) | 7 (23) | 6 (21) | 5 (13) | 6 (13) | 0 (0) |
| ARQ/AHQ | 5 (10) | 5 (12) | 1 (3) | 1 (3) | 1 (3) | 1 (2) | 0 (0) |
| ARQ/ARQ | 2 (5) | 0 (0) | 0 (0) | 0 (0) | 0 (0) | 0 (0) | 0 (0) |
| VRQ/ARR | 12 (25) | 11 (27) | 8 (26) | 3 (10) | 3 (8) | 3 (6) | 0 (0) |
| VRQ/AHQ | 1 (2) | 0 (0) | 0 (0) | 0 (0) | 0 (0) | 0 (0) | 0 (0) |
| VRQ/ARQ | 6 (13) | 3 (7) | 0 (0) | 0 (0) | 0 (0) | 0 (0) | 0 (0) |
| VRQ/VRQ | 0 (0) | 0 (0) | 0 (0) | 0 (0) | 0 (0) | 0 (0) | 0 (0) |
| Missing | 0 (0) | 0 (0) | 0 (0) | 0 (0) | 0 (0) | 0 (0) | 0 (0) |
| Total | 48 (100) | 41 (100) | 31 (100) | 29 (100) | 38 (100) | 47 (100) | 23 (100) |

|  | Flock E: # ewes (%) per year | | | | | | |
| --- | --- | --- | --- | --- | --- | --- | --- |
| Genotype | 2000 | 2001 | 2002 | 2003 | 2004 | 2005 | 2006 |
| ARR/ARR | 12 (8) | 20 (13) | 27 (18) | 42 (27) | 71 (41) | 109 (53) | 151 (65) |
| AHQ/ARR | 1 (1) | 1 (1) | 0 (0) | 0 (0) | 0 (0) | 0 (0) | 0 (0) |
| AHQ/AHQ | 0 (0) | 0 (0) | 0 (0) | 0 (0) | 0 (0) | 0 (0) | 0 (0) |
| ARH/ARR | 0 (0) | 0 (0) | 0 (0) | 0 (0) | 0 (0) | 3 (2) | 3 (1) |
| ARQ/ARR | 51 (34) | 58 (36) | 68 (44) | 73 (47) | 76 (44) | 72 (35) | 70 (30) |
| ARQ/AHQ | 3 (2) | 0 (0) | 0 (0) | 0 (0) | 0 (0) | 0 (0) | 0 (0) |
| ARQ/ARQ | 46 (31) | 44 (28) | 34 (22) | 19 (13) | 11 (6) | 7 (3) | 4 (2) |
| VRQ/ARR | 11 (7) | 15 (9) | 10 (6) | 11 (7) | 9 (5) | 9 (4) | 3 (1) |
| VRQ/AHQ | 0 (0) | 0 (0) | 0 (0) | 0 (0) | 0 (0) | 0 (0) | 0 (0) |
| VRQ/ARQ | 21 (14) | 18 (11) | 12 (8) | 8 (5) | 6 (3) | 4 (2) | 2 (1) |
| VRQ/VRQ | 4 (3) | 3 (2) | 2 (1) | 2 (1) | 0 (0) | 0 (0) | 0 (0) |
| Missing | 0 (0) | 0 (0) | 1 (1) | 0 (0) | 2 (1) | 1 (1) | 1 (0) |
| Total | 149 (100) | 159 (100) | 154 (100) | 155 (100) | 175 (100) | 205 (100) | 234 (100) |

|  | Flock F: # ewes (%) per year | | | | |
| --- | --- | --- | --- | --- | --- |
| Genotype | 2000 | 2001 | 2002 | 2003 | 2004 |
| ARR/ARR | 9 (30) | 11 (29) | 16 (35) | 6 (30) | 6 (32) |
| AHQ/ARR | 0 (0) | 1 (3) | 1 (2) | 1 (5) | 1 (5) |
| AHQ/AHQ | 0 (0) | 0 (0) | 0 (0) | 0 (0) | 0 (0) |
| ARQ/ARR | 0 (0) | 3 (8) | 4 (9) | 3 (15) | 2 (10) |
| ARQ/AHQ | 0 (0) | 0 (0) | 0 (0) | 0 (0) | 0 (0) |
| ARQ/ARQ | 0 (0) | 0 (0) | 0 (0) | 0 (0) | 0 (0) |
| VRQ/ARR | 18 (60) | 21 (57) | 24 (52) | 10 (50) | 10 (53) |
| VRQ/AHQ | 1 (3) | 1 (3) | 1 (2) | 0 (0) | 0 (0) |
| VRQ/ARQ | 1 (4) | 0 (0) | 0 (0) | 0 (0) | 0 (0) |
| VRQ/VRQ | 1 (3) | 0 (0) | 0 (0) | 0 (0) | 0 (0) |
| Missing | 0 (0) | 0 (0) | 0 (0) | 0 (0) | 0 (0) |
| Total | 30 (100) | 37 (100) | 46 (100) | 20 (100) | 19 (100) |

**Table S2**: Detailed information on the 59 scrapie cases included in the study. Scrapie positive test result = 1, scrapie negative test result = 0, Y = young (< 1 year old), p.m. = post mortem examination, M = missing data, X = not present.

|  |  |  |  | Tonsil biopsy | | | | | |  |  | Post portem | |
| --- | --- | --- | --- | --- | --- | --- | --- | --- | --- | --- | --- | --- | --- |
| Flock | Case | Born | Genotype | 2000 | 2001 | 2002 | 2003 | 2004 | 2005 | 2006 | Death/culling | tonsils | obex |
|  |  |  |  |  |  |  |  |  |  |  |  |  |  |
| A | 1 | 4-1998 | VRQ/ARQ | 1 | X | X | X | X |  |  | 6-2001 | 1 | 1 |
|  |  |  |  |  |  |  |  |  |  |  |  |  |  |
| B | 1 | 3-1997 | VRQ/ARQ | 1 | X | X | X | X | X | X | 1-2001 | 1 | 1 |
|  | 2 | 3-1996 | ARQ/ARQ | p.m. | X | X | X | X | X | X | 6-2000 | 1 | 1 |
|  | 3 | 3-1999 | VRQ/ARQ | 0 | 1 | X | X | X | X | X | 1-2002 | M | 1 |
|  | 4 | 3-2000 | VRQ/ARQ | Y | 1 | X | X | X | X | X | 2-2002 | 1 | 1 |
|  | 5 | 3-1999 | VRQ/ARQ | 0 | 0 | X | X | X | X | X | 8-2001 | 1 | 1 |
|  | 6 | 3-1999 | VRQ/ARQ | 0 | 0 | 1 | 1 | X | X | X | 7-2003 | 1 | 1 |
|  |  |  |  |  |  |  |  |  |  |  |  |  |  |
| C | 1 | 3-1999 | ARQ/ARQ | 1 | X | X | X | X | X | X | 3-2001 | 1 | 1 |
|  | 2 | < 1998 | ARQ/ARQ | 1 | X | X | X | X | X | X | 9-2000 | 1 | 1 |
|  | 3 | < 1999 | ARQ/ARQ | 1 | X | X | X | X | X | X | 6-2001 | 1 | 1 |
|  | 4 | < 1999 | ARQ/ARQ | 1 | X | X | X | X | X | X | 9-2000 | 1 | 1 |
|  | 5 | 3-1998 | ARQ/ARQ | 1 | X | X | X | X | X | X | 7-2001 | M | M |
|  | 6 | < 2000 | ARQ/ARQ | p.m. | X | X | X | X | X | X | 6-2000 | 1 | 0 |
|  | 7 | < 1998 | ARQ/ARQ | p.m. | X | X | X | X | X | X | 6-2000 | 1 | 1 |
|  | 8 | < 1999 | ARQ/ARQ | p.m. | X | X | X | X | X | X | 6-2000 | 1 | 1 |
|  | 9 | 3-1999 | ARQ/ARQ | 0 | 1 | M | X | X | X | X | 9-2002 | M | M |
|  | 10 | 3-1999 | ARQ/ARQ | 0 | 1 | 1 | X | X | X | X | 7-2002 | 1 | 1 |
|  | 11 | 3-1999 | ARQ/ARQ | 0 | 1 | X | X | X | X | X | 5-2002 | 1 | 1 |
|  | 12 | 3-1999 | ARQ/ARQ | 0 | 1 | M | X | X | X | X | 1-2003 | 1 | 1 |
|  | 13 | 3-2000 | ARQ/ARQ | Y | 1 | 1 | X | X | X | X | 1-2003 | 1 | 1 |
|  | 14 | 3-2000 | ARQ/ARQ | Y | 1 | 1 | X | X | X | X | 2-2003 | 1 | 1 |
|  | 15 | 3-1998 | ARQ/ARQ | 0 | 1 | X | X | X | X | X | 10-2001 | 1 | 1 |
|  | 16 | 3-1999 | ARQ/ARQ | 0 | 0 | 1 | X | X | X | X | 1-2003 | 1 | 1 |
|  | 17 | 3-1999 | ARQ/ARQ | 0 | 0 | 1 | X | X | X | X | 1-2003 | 1 | 1 |
|  | 18 | 3-2000 | ARQ/ARQ | Y | 0 | 1 | X | X | X | X | 5-2003 | 1 | 1 |
|  | 19 | 3-2000 | ARQ/ARQ | Y | 0 | 1 | 1 | X | X | X | 6-2003 | 1 | 1 |
|  | 20 | 3-2000 | ARQ/ARQ | Y | 0 | 1 | X | X | X | X | 1-2003 | 1 | 1 |
|  | 21 | 3-2000 | ARQ/ARQ | Y | 0 | M | X | X | X | X | 10-2002 | 1 | 1 |
|  | 22 | 3-1999 | ARQ/ARQ | 0 | 0 | M | X | X | X | X | 12-2002 | 1 | 1 |
|  | 23 | 3-1999 | ARQ/ARQ | 0 | 0 | M | X | X | X | X | 1-2003 | 1 | 1 |
|  | 24 | 3-1998 | ARQ/ARQ | 0 | 0 | 0 | X | X | X | X | 5-2003 | 1 | 1 |
|  | 25 | 3-2000 | ARQ/ARQ | Y | 0 | 0 | 1 | X | X | X | 11-2003 | 1 | 1 |
|  | 26 | 3-2000 | ARQ/ARQ | Y | 0 | 0 | 1 | X | X | X | 6-2003 | 1 | 1 |
|  | 27 | 3-2000 | ARQ/ARQ | Y | 0 | 0 | 1 | X | X | X | 2-2004 | 1 | 1 |
|  | 28 | <2000 | ARQ/ARQ | 0 | 0 | 0 | 1 | X | X | X | 3-2004 | 1 | 1 |
|  | 29 | 3-2000 | ARQ/ARQ | Y | 0 | 0 | 0 | X | X | X | 12-2003 | 1 | 1 |
|  | 30 | 3-1999 | ARQ/ARQ | 0 | 0 | 0 | 0 | 0 | X | X | 2-2005 | 0 | 1 |
|  | 31 | 3-2000 | ARQ/ARQ | Y | 0 | 0 | 0 | 0 | 0 | M | 10-2006 | 1 | 0 |
|  |  |  |  |  |  |  |  |  |  |  |  |  |  |
| D | 1 | 2-1998 | VRQ/ARQ | 1 | X | X | X | X | X | X | 1-2001 | 1 | 1 |
|  | 2 | 2-1998 | VRQ/ARQ | 1 | 1 | X | X | X | X | X | 9-2001 | 1 | 1 |
|  | 3 | 1-1999 | VRQ/ARQ | 1 | 1 | X | X | X | X | X | 9-2001 | 1 | 1 |
|  | 4 | 2-1998 | VRQ/ARQ | p.m. | X | X | X | X | X | X | 7-2000 | 1 | 1 |
|  | 5 | 2-1998 | VRQ/ARQ | p.m. | X | X | X | X | X | X | 7-2000 | 1 | 0 |
|  | 6 | 2-1998 | VRQ/ARQ | 0 | p.m. | X | X | X | X | X | 6-2001 | 1 | 1 |
|  | 7 | 2-1998 | VRQ/ARR | 0 | 0 | 0 | X | X | X | X | 7-2002 | 0 | 1 |
|  | 8 | 2-1998 | VRQ/ARR | 0 | 0 | 0 | X | X | X | X | 4-2003 | 0 | 1 |
|  | 9 | 2-1998 | VRQ/ARR | 0 | 0 | M | X | X | X | X | 4-2003 | 0 | 1 |
|  |  |  |  |  |  |  |  |  |  |  |  |  |  |
| E | 1 | 3-1997 | VRQ/ARQ | 1 | 1 | X | X | X | X | X | 3-2002 | 1 | 1 |
|  | 2 | 3-1998 | VRQ/VRQ | 1 | 1 | X | X | X | X | X | 1-2002 | M | 1 |
|  | 3 | 1-1999 | VRQ/ARQ | 1 | 1 | X | X | X | X | X | 1-2002 | 1 | 1 |
|  | 4 | 3-1999 | VRQ/ARQ | 1 | 1 | X | X | X | X | X | 2-2002 | 1 | 1 |
|  | 5 | 3-1997 | ARQ/ARQ | 0 | M | 1 | p.m. | X | X | X | 9-2003 | 1 | 1 |
|  |  |  |  |  |  |  |  |  |  |  |  |  |  |
| F | 1 | 3-1999 | VRQ/ARQ | 1 | X | X | X | X |  |  | 2-2001 | 1 | 1 |
|  | 2 | 3-1997 | VRQ/VRQ | p.m. | X | X | X | X |  |  | 9-2000 | 1 | 1 |
|  | 3 | 3-1999 | VRQ/ARR | 0 | 0 | X | X | X |  |  | 5-2002 | 0 | 1 |
|  | 4 | 3-1999 | VRQ/ARR | 0 | 0 | 0 | X | X |  |  | 2-2003 | 0 | 1 |
|  | 5 | 3-1999 | VRQ/ARR | 0 | 0 | M | X | X |  |  | 2-2003 | 0 | 1 |
|  | 6 | 3-1999 | VRQ/ARR | 0 | 0 | 0 | p.m. | X |  |  | 9-2003 | 0 | 1 |
|  | 7 | 3-1999 | VRQ/ARR | 0 | 0 | 0 | 0 | X |  |  | 9-2004 | 0 | 1 |

**Table S3.** Realizations of the chance process described by the conditional probability $P\left(x=a|x>a-1\right)$**.**

| *A* | 1 | 2 | 3 | 4 | 5 | 6 | 7 |
| --- | --- | --- | --- | --- | --- | --- | --- |
| *N(a)* | 56 | 50 | 29 | 18 | 11 | 10 | 2 |
| *m(a)* | 3 | 8 | 8 | 0 | 0 | 1 | 0 |

**Table S4.** Point estimates and confidence interval boundaries for the infection probabilities of different genotypes based on field data.

| Flock | Cohort | Genotype γ | number of animals | number of scrapie cases | point estimate for infection probability *p*_γ_ | confidence bounds for infection probability *p*_γ_ |
| --- | --- | --- | --- | --- | --- | --- |
| B | 1999 | VRQ/ARQ ARQ/ARQ ARQ/ARR | 6  11  10 | 3  0  0 | 0.5  0.0  0.0 | (0.118, 0.882)  (0.0, 0.238)  (0.0, 0.259) |
| C | 1999 & 2000 | ARQ/ARQ ARQ/ARR | 56  14 | 20  0 | 0.416  0.0 | (0.273, 0.574)  (0.0, 0.193) |
| D | 1998 | VRQ/ARQ VRQ/ARR | 5  4 | 5  3 | 1.0  0.244 | (0.549, 1.0)  (0.102, 0.619) |
| E | 1997 | VRQ/ARQ  ARQ/ARQ  ARQ/ARR | 10  20  17 | 3  1  0 | 0.163  0.0253  0.0 | (0.0340, 0.411)  (0.00063, 0.249)  (0.0, 0.0846) |
